# The estimation and use of predictions for the assessment of model performance using large samples with multiply imputed data

**DOI:** 10.1002/bimj.201400004

**Published:** 2015-01-29

**Authors:** Angela M Wood, Patrick Royston, Ian R White

**Affiliations:** 1 Department of Public Health and Primary Care, Strangeways Research Laboratory, University of Cambridge, Worts CausewayCambridge CB1 8RN, UK; 2 MRC Clinical Trials Unit at UCL, Aviation House125 Kingsway, London WC2B 6NH, UK; 3 MRC Biostatistics Unit, Cambridge Institute of Public Health, Forvie Site, Robinson Way, Cambridge Biomedical CampusCambridge CB2 0SR, UK

**Keywords:** Measures of model performance, Missing data, Model validation, Multiple imputation, Prediction models, Rubin's rules

## Abstract

Multiple imputation can be used as a tool in the process of constructing prediction models in medical and epidemiological studies with missing covariate values. Such models can be used to make predictions for model performance assessment, but the task is made more complicated by the multiple imputation structure. We summarize various predictions constructed from covariates, including multiply imputed covariates, and either the set of imputation-specific prediction model coefficients or the pooled prediction model coefficients. We further describe approaches for using the predictions to assess model performance. We distinguish between *ideal model performance* and *pragmatic model performance*, where the former refers to the model's performance in an ideal clinical setting where all individuals have fully observed predictors and the latter refers to the model's performance in a real-world clinical setting where some individuals have missing predictors. The approaches are compared through an extensive simulation study based on the UK700 trial. We determine that measures of ideal model performance can be estimated within imputed datasets and subsequently pooled to give an overall measure of model performance. Alternative methods to evaluate pragmatic model performance are required and we propose constructing predictions either from a second set of covariate imputations which make no use of observed outcomes, or from a set of partial prediction models constructed for each potential observed pattern of covariate. Pragmatic model performance is generally lower than ideal model performance. We focus on model performance within the derivation data, but describe how to extend all the methods to a validation dataset.

## 1. Introduction

The derivation and assessment of prediction models in data with missing covariate values present challenges. We (Wood et al., 2008; White et al., 2011) and others (Ambler et al., 2007; Vergouw et al., 2010; Carpenter and Kenward, 2013) have previously described multiple imputation methods to deal with missing values for prediction model derivation. In this paper we consider the estimation and use of predictions in postestimation procedures in multiply imputed data.

The standard multiple imputation procedure (Rubin, 1987; Schafer, 1997) replaces missing covariate data with values drawn from a set of specified imputation models based on the observed relationships between the covariates and outcome, typically under a missing at random (MAR) assumption (Little and Rubin, 1987). A key feature of the procedure is that it outputs a number, say *M*, of imputed datasets. A prediction model is then fitted to each imputed dataset to produce imputation-specific regression coefficients, which can be averaged using “Rubin's rules” (Rubin, 1987) to provide pooled regression coefficients. To extract predictions from such a prediction model and multiply imputed data, either the sets of imputation-specific regression coefficients or the pooled regression coefficients could be used, and we must decide how to handle individuals with multiply imputed covariates. Thus there are numerous combinations for constructing a set of predictions, including imputation-specific predictions (*M* predictions for each individual) and pooled predictions (an averaged prediction over imputed datasets for each individual). The differences between them and their advantages and drawbacks are unclear.

Predictions are required for assessing model performance through the estimation of various measures: for example, mean squared prediction error (MSPE), *R*^2^ measures, area under the receiver operating curve (AUROC) and goodness-of-fit measures (Hosmer and Lemeshow, 1989, Chapter 5). The multiple imputation procedure presents specific challenges for such assessments (Marshall et al., 2009). For example, *M* model performance measures can be estimated from the imputation-specific predictions and then pooled using Rubin's rules, as previously recommended (Marshall et al., 2009; Vergouwe et al., 2010; White et al., 2011; Moons et al., 2012b). Alternatively, an overall measure of model performance can be estimated directly from a set of pooled predictions, but the consequences of this approach remain unclear (Vergouwe et al., 2010). In addition, the impact on model performance measures from using predictions constructed from imputed covariates that have been partly derived from the observed outcome requires consideration. Previous work (Schafer, 1997; Moons et al., 2006; Sterne et al., 2009; White et al., 2011) emphasizes the importance of including the outcome in imputation models in order to maintain observed relationships between covariates and the outcome as required for regression modeling. However, since model performance measures are defined as functions of the observed outcomes and predictions, we hypothesize that such measures may be optimistic when the predictions are constructed from a set of imputed covariates partly derived from the observed outcomes.

Model performance measures aim to quantify the usefulness of a prediction model for a future clinical setting, usually under the assumption that individuals in the future clinical setting have fully observed predictors. For example, evaluations of the QRISK2 cardiovascular (Collins and Altman, 2010) and QCancer (Colorectal) (Collins and Altman, 2012) prediction models were performed after applying multiple imputation to replace any missing predictors. Here, the multiple imputation procedure is not advocated for use in the clinical setting, especially since it requires knowledge about the outcome (which of course is not yet available), but instead acts as an initial statistical tool to enable model performance evaluation. Such an evaluation assumes that individuals in the future clinical setting have fully observed predictors. Alternatively, it is possible to quantify the usefulness of a prediction model in a more realistic future clinical setting where individuals may have some missing predictors. For example, an evaluation of the earlier QRISK cardiovascular prediction model (Collins and Altman, 2009) was performed using observational data with missing predictors replaced with reference values matched for age and sex. Here, the imputation procedure *was* advocated for use in the clinical setting (Hippisley-Cox et al., 2007) and was considered as part of the full risk prediction algorithm to be evaluated. Such an evaluation assumes that individuals in the future clinical setting have similar patterns of missing covariates to the observational data used in the evaluation. In this paper we propose two new generic strategies for this latter evaluation.

The structure of this paper is as follows. We first introduce methods for estimating imputation-specific predictions and pooled predictions, using multiply imputed covariates and using regression coefficients from a prediction model constructed on multiply imputed data (Section 1987). We focus on the three main issues introduced above: (1) use of imputation-specific or pooled regression coefficients for estimating predictions; (2) using predictions to estimate model performance measures and (3) the evaluation of the model performance in the context of future clinical settings where predictor information may be fully observed or partly missing. We present details of a simulation study to investigate the behavior of common measures of model performance using the prediction methods we describe (Section 100). Results of the simulation study are presented (Section 220). We then explore assessment of calibration using data from the UK700 trial (Section 260). We conclude by discussing the findings of the paper and making recommendations for applied analyses (Section 270). The simulations considered in this paper relate to using predictions to assess performance in the derivation dataset and issues regarding validation are left for discussion.

## 2. Multiple imputation and predictions

### 2.1. Notation

Here we introduce the notation used throughout the paper. Let the dataset on which the prediction model is derived be the derivation dataset with *n* individuals. We assume individuals have some missing covariate values but their outcomes are fully observed.

We assume a suitable initial multiple imputation procedure (Schafer, 1997; White et al., 2011) has been performed using all covariate and outcome variables to replace the missing covariate values with *M* independent sets of values. We use the superscript (*k*) to refer to the *k*th imputation in the derivation dataset, where *k* = 1, …, *M*. The prediction model fitted to the *k*th imputed derivation dataset can be written as 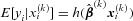 where *y_i_* represents the observed outcome variable for individual *i* (for *i* = 1,…, *n*), *h*(.) represents a suitable inverse link function for a generalized linear model (noting that an extension to the Cox proportional hazards model is relatively straightforward), 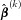 represents the *k*th vector of estimated *imputation-specific* regression coefficients and 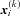 represents the vector of observed and any imputed covariates from the *k*th imputation for individual *i*. Pooled regression coefficients are obtained using Rubin's rules (Rubin, 1987) to give 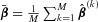.

We also introduce a second set of imputed covariates, denoted by 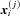 where superscript (*j*) refers to the *j*th imputation for *j* = 1,…, *M_2_*, and *i* = 1,…, *n*. In this paper, we specifically consider the case where 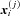 are multiple imputations from a second imputation procedure with the same variables as the initial imputation approach but with the outcome variable excluded from the imputation models. This imputation procedure is useful to imitate a future clinical setting in which individuals have unknown outcome and partly missing predictors. More generally, 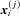 can denote imputed covariates from any imputation model where the (*j)* and (*k*) indices represent distinct imputations.

We let 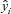 denote individual *i*'s pooled predicted outcome (defined in subsection 50). A performance measure for the prediction model (e.g., MSPE, *R*^2^, AUROC) may be calculated using observed and predicted outcomes. We denote by 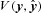 a model performance measure of interest, where 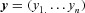 and 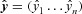.

### 2.2. “Ideal model performance” and “Pragmatic model performance”

We distinguish between two model performance estimators to quantify the usefulness of a prediction model in future clinical settings:

*Ideal model performance* refers to the model's performance in an ideal future clinical setting where all individuals have fully observed predictors;

*Pragmatic model performance* refers to the model's performance in a realistic future clinical setting where some individuals may have partly missing predictors.

In Sections 50–90 we explain how these model performance measures can be estimated. For measures of pragmatic model performance, a strategy for handling missing predictors in the future clinical setting must be chosen and is evaluated as part of the risk prediction algorithm. We assume the missing data pattern in the derivation dataset is representative of the future clinical setting.

### 2.3. Estimating and using predictions in multiply imputed data

Possible imputation-specific predictions (P1, P4, and P7) and pooled predictions (P2, P3, P5, P6, P8, and P9) constructed using imputation-specific regression coefficients 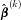 are defined in Table 1 and described in more detail in the subsections below. Alternative predictions replace 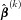 with the pooled regression coefficients 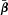.

**Table 1:** A summary of possible imputation-specific and pooled predictions from a model fitted to multiply imputed data with imputation-specific regression coefficients 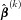. For a linear model where *h*(.) is the identity function, P2 = P3, P5 = P6, and P8 = P9. Alternative predictions use 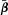 in place of 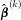

Covariate values	Imputation-specific predictions	Pooled predictions
Individuals with fully observed covariates ***x**_i_*	(P1) 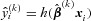	(P2) 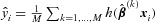 or (P3) 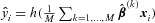
Individuals with any imputed covariates 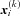 *k* = 1,…, *M*	(P4) 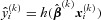	(P5) 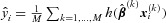 or (P6) 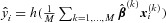
Individuals with any imputed covariates 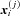 *j* = 1,…, *M*_2_	(P7) 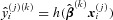	(P8) 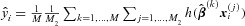 or (P9) 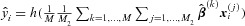

**Figure 1:**
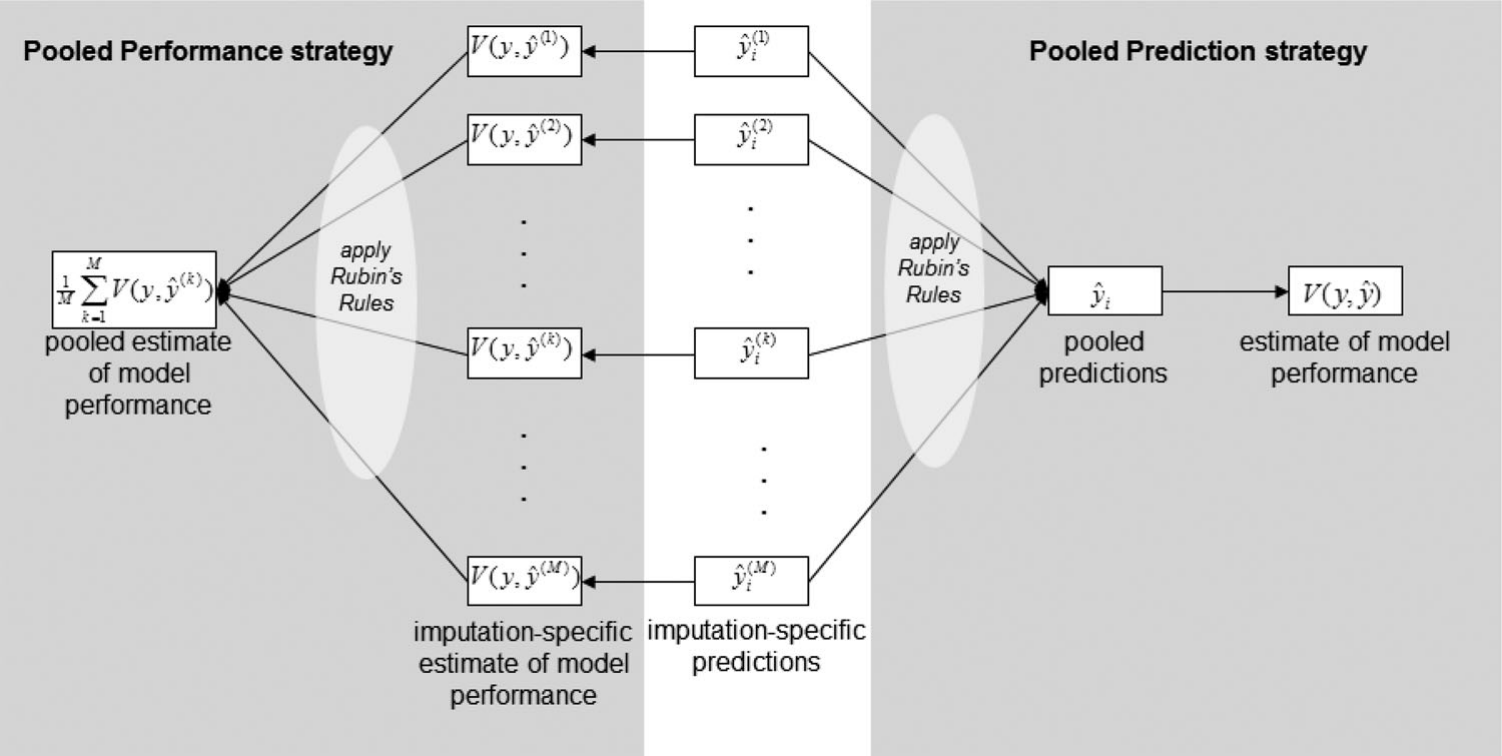
Illustration to show how measures of model performance can be estimated from multiply imputed data.

Imputation-specific predictions may be used to estimate imputation-specific performance measures 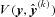 or 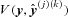 which can be subsequently pooled over imputations using Rubin's rules (Rubin, 1987) to give 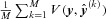 or 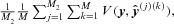 respectively. We call this strategy the “*pooled performance*” strategy, as Rubin's rules are applied to the set of imputation-specific estimates of model performance, as shown in Fig.1. Alternatively, an overall performance measure may be directly calculated using the pooled predictions. We call this strategy the “*pooled predictions*” strategy, as Rubin's rules are applied to the set of imputation-specific predictions, as shown in Fig.1.

#### 2.3.1. Predictions P1–P3 based on fully observed covariates

Imputation-specific predictions P1 are obtainable for individuals with fully observed covariates. P1 or its corresponding linear predictor may be averaged over imputations using Rubin's rules to give pooled predictions P2 and P3, respectively. Since Rubin's rules are based on Normal theory, it is likely that the pooled linear predictor is more accurately estimated than the pooled prediction, since the posterior distribution of the linear predictor is likely to be closer to the Normal distribution (Schafer, 1997; Marshall et al., 2009). Predictions P1–P3 may be used to crudely estimate measures of ideal model performance among individuals with fully observed covariates (noting that the prediction model is still fitted to all individuals). For example, see van de Laar et al. (2014).

#### 2.3.2. Predictions P4–P6 based on multiply imputed covariates 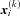


Imputation-specific predictions P4 are obtainable for individuals with imputed covariates 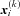. Imputation-specific predictions may be averaged over imputations using Rubin's rules to give pooled predictions P5 or imputation-specific linear predictors may be averaged over imputations using Rubin's rules and then transformed to give pooled predictions P6. Earlier, we raised the concern about evaluating model performance using predicted outcomes constructed from imputed covariates 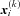, which are partly derived on the observed outcome. More specifically, we note that in calculating P5 and P6, the between-imputation variability in 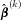 and 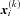 is averaged out (and thus eliminated before assessing model performance), resulting in a single deterministic prediction, derived from observed ***x*** and ***y***. A performance measure based on pooled predictions P5 or P6 may thus be optimistic, especially when ***y*** is a strong predictor in the multiple imputation procedure. We thus do not advocate the use of P5 or P6 in assessing model performance.

We propose predictions P4 (which reduces to P1 for individuals with fully observed covariates) may be used to estimate measures of ideal model performance on all individuals. This approach has been frequently adopted in the literature (Clark and Altman, 2003; Steyerberg et al., 2008; Collins and Altman, 2009, 2010, 2012; O'Mahony et al., 2014).

#### 2.3.3. Predictions P7–P9 based on multiply imputed covariates 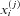


Imputation-specific predictions P7 are obtainable using the second set of *M*_2_ imputed covariates. Since the imputation indexes (*j*) and (*k*) are distinct, averaging over both *j* and *k* is required to obtain pooled predictions as given by P8 and P9. We note that in calculating P8 and P9 the between-imputation variability in 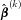 and 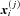 is averaged out, resulting in single deterministic predictions. Explicitly, the observed outcome is used in the derivation of 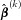 but not 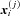.

Predictions P7–P9 (which reduce to P1–P3 for individuals with fully observed covariates) may be used to estimate measures of pragmatic model performance, under the assumption that missing predictors in the future clinical setting will be handled using multiply imputation in the same way. Model performance based on P7 will be poorer than that from P8 or P9 because of the additional between-imputation variability induced by the second imputation procedure. To our knowledge, model performance measures based on P7–P9 have not been used in the literature.

### 2.4. Partial prediction models

We propose a further strategy to evaluate pragmatic model performance based on a set of partial prediction models. Instead of constructing and evaluating a single prediction model which includes all covariates of interest, we construct and evaluate a set of prediction models, where each model contains a distinct set of covariates for each subset of observed covariates. For example, in a setting with three covariates denoted by X1, X2, and X3, where X1 is always observed but X2 and X3 may be missing, there exist three potential partial prediction models with covariate sets {X1}, {X1, X2}, and {X1, X3}, and the full prediction model with covariate set {X1, X2, X3}. Predictions are extracted from the partial prediction model with covariate set {X1} for individuals with observed X1 and missing X2 and X3; predictions are extracted from the partial prediction model with covariate set {X1, X2} for individuals with observed X1 and X2 and missing X3; predictions are extracted from the partial prediction model with covariate set {X1, X3} for individuals with observed X1 and X3 and missing X2 and predictions are extracted from the full prediction model for individuals with observed X1, X2, and X3. We propose fitting the full and each partial prediction model to all individuals in the multiply imputed derivation dataset to give model-specific estimates 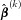 and 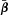, from which predictions P1–P3, based on fully observed covariates, may be extracted and used to estimate measures of pragmatic model performance. We note that the predictions are derived on observed covariates only. For implementation, each partial prediction model needs the same careful construction as required for a single prediction model (Wood et al., 2008; Moons et al., 2012b). In datasets with many missing covariates and distinct patterns, constructing many partial prediction models may be impractical.

## 3. Simulation approach

### 3.1. UK700 dataset

We use simulated data from the UK700 trial to illustrate and compare the different approaches for extracting and using predictions from multiply imputed data. The UK700 trial was a multicentre randomized controlled trial conducted in four inner-city areas of the UK (Burns et al., 1999). The main trial findings have been previously reported (Burns et al., 1999). Participants were aged 18–65 with a diagnosed psychotic illness and at least two previous psychiatric hospital admissions, the most recent within the previous 2 years. Such patients were typically managed in the community by a case manager. A total of 708 participants were randomly allocated to a case manager with a case load of either 30–35 patients (standard case management) or 10–15 patients (intensive case management). The trial outcomes and baseline characteristics used in this present paper are summarized in Table 2.

**Table 2:** Summary of variables in the UK700 trial

Variable	Data type	Code	Number of individuals with observed values	Mean (SD) or *n* (%)
Outcome variables recorded at two years				
Comprehensive psychopathological rating scale	*Continuous*	cprs	595	2.65 (0.87)a)
(Dis)satisfaction with case management	*Continuous*	sat	490	16.90 (4.78)a)
Baseline variables used as covariates in simulated prediction model				
Comprehensive psychopathological rating scale	*Continuous*	cprs0	705	2.73 (0.82)a)
Centre	*Categorical*	centre	708	
		St George's		196 (28%)
		Manchester		158 (22%)
		St Mary's		201 (39%)
		King's		153 (22%)
Total disability score	*Continuous*	distot	659	−0.07 (0.81)a)
Time from onset of psychosis to study entry (months)	*Continuous*	onset	705	4.62 (0.98)a)
Age (years)	*Continuous*	age	708	38.29 (11.64)
Sex	*Binary*	sex	708	
		female		304 (43%)
		male		404 (57%)
Outpatient status at recruitment	*Binary*	status in hospital outpatient	707	
				289 (41%)
				418 (59%)
Other baseline variables				
Missing father's occupation at birth	*Binary*	occgp	708	
		observed		576 (81%)
		missing		132 (19%)
(Dis)satisfaction with case management	*Continuous*	sat0	410	18.86 (4.83)

a) Mean (SD) of the log-transformed values.

### 3.2. Simulation procedure

The aim of our simulation studies is to compare and assess the merits of the approaches described in Section 1987. Our simulation strategy is as follows:

1. Start with *n* = 708 and a set of complete covariates denoted by ***x***.
2. Simulate the continuous or binary outcome variable ***y*** from a pre-defined linear or logistic model.
3. Induce missing data in ***x*** under a variety of missing data patterns.
4. Multiply impute missing data in ***x***.
5. Fit the prediction model and the partial prediction models to the multiply imputed data.
6. Extract the obtainable imputation-specific and pooled predictions as defined in Table 1 and calculate model performance measures.
7. Repeat steps 2–6 1000 times, and compare results across the simulations.

Results are presented in Section 220 for 12 simulation scenarios, which differ according to the outcome variable (continuous or binary), amount and type of missing data and strength of predictors. These scenarios and each of the strategy steps are described in detail below.

### 3.3. Complete data: The UK700 trial

The simulations are based on the comprehensive psychopathology rating scale (CPRS) outcome at 2 years. We used seven baseline variables from Table 2: baseline CPRS (cprs0), centre, onset, total disability, age, sex, and outpatient status. Due to their skewed distributions, we used log-transformations of cprs+1, cprs0+1, onset, and distot. The outcome and covariates were chosen because they are reasonably complete (up to 20% missing) and all covariates are univariately significant predictors of cprs at 2 years at the 5% level.

A single completed dataset was formed by imputing missing values in variables cprs, cprs0, distot, onset, and status using a single imputation with Stata's mi impute command. Note the method used to impute the missing values at this stage has little impact on the simulation results. The resulting completed dataset forms the basis of our simulation studies.

### 3.4. Simulation models for outcome *y*

Outcome values were simulated from a linear model and a logistic model, each derived from the completed UK700 dataset. The linear model was:

Linear model

1. log(crps + 1) = 1.64 + 0.34log(cprs0 + 1) − 0.01age + 0.11log(onset) + 0.20log(distot) − 0.19sex + 0.15status + 0.06centre2 + 0.30centre3 + 0.0008centre4 + N(0,0.61)

This model had *R*^2^ = 0.22 in the original UK700 dataset. To explore the impact of stronger predictors, we repeated the simulations with the regression coefficient for log(crps0+1) increased from 0.34 to 0.68.

Two logistic models were considered for a dichotomized cprs outcome using two cut-off values of cprs>25 and cprs>40 to produce two binary outcomes with prevalence 26.5% and 8.6%. The logistic models used were

Logistic models

1. Logit(P(cprs>25)) = −3.20 + 0.75 log(cprs0 + 1) − 0.03age + 0.25log(onset) + 0.44 log(distot) − 0.28sex − 0.14status − 0.29centre2 + 0.91centre3 + 0.16centre4
2. Logit(P(cprs>40)) = − 6.54 + 1.13 log(cprs0 + 1) − 0.04age + 0.37log(onset) + 0.51 log(distot) − 0.50sex − 0.07status − 0.63centre2 + 1.55centre3 + 0.86centre4.

The second logistic model was constructed to allow us to explore the impact of a rare outcome on the choice of averaging the linear predictors or the probabilities, since the latter would be expected to be non-normally distributed. These models had AUROC of 0.73 and 0.79, respectively, in the original UK700 dataset.

Thus, each complete simulated dataset consists of completed values for baseline CPRS, centre, total disability, time from onset of psychosis to study entry, age, sex, and outpatient status, and simulated values for continuous cprs and dichotomized cprs. The observed outcome values were no longer used.

### 3.5. Inducing missing data

We explored the following patterns of missing data:

#### 3.5.1. Independent MCAR

We deleted a completely random 30% of the data in the variable cprs0 and deleted a completely random 10% of the data in the variables onset and distot, independent of the data values and independent of missingness in other variables.

#### 3.5.2. Monotone MCAR

We created three random subgroups of size 10% of the data. We deleted data on distot in the first subgroup, data on onset in the first two subgroups, and data on cprs0 in all three subgroups. This is the same missing data pattern as for MAR (below) and enables a clear comparison of prediction approaches between the MCAR and MAR assumptions.

#### 3.5.3. Monotone MAR

We constructed a monotone MAR missing data model based on a logistic model for the probability of missing values in a further variable, father's occupation (occgp), similar to the procedure used in Wood et al. (2008). Father's occupation was missing in 19% of patients in the study. Missing data in covariates cprs0, onset, and distot were imposed in the following stages to induce 30% missingness in cprs0:

1. Construct a logistic model using the outcome defined as missing occgp and covariates age, sex and status. Impose missing data in cprs0, onset, and distot for individuals with fitted probabilities falling in the top tenth of probabilities. This step induces 10% MAR missingness in cprs0, onset, and distot.
2. Construct a logistic model for missing occgp regressing on variables distot, age, sex, and status and excluding individuals with missing values imposed by stage 1. Impose missing data in cprs0 and onset for individuals with fitted probabilities falling in the top ninth of probabilities. This step induces further 10% MAR missingness in cprs0 and onset.
3. Construct a logistic model for missing occgp regressing on variables onset, distot, age, sex, and status and excluding individuals with missing values imposed by stages 1 & 2. Impose missing data in cprs0 for individuals with fitted probabilities falling in the top eighth of probabilities. This step induces further 10% MAR missingness in cprs0.

Centre was excluded from the missing data model because it was highly predictive of missing data in occgp and we wanted to produce a MAR pattern more equally dependent on the other covariates. This strategy produced the same pattern of missing data as in 3.5.2 above, but with a different mechanism. An identical strategy but with cut-offs of the top fifth, top quarter, and top third for steps (1)–(3), respectively, was employed to induce 60% missing at random in cprs0.

### 3.6. Multiple imputation using chained equations

Multiple imputation was performed using the mi impute chained (pmm) command in Stata 12 (StataCorp, 2011), which uses the iterated chained equations approach (van Buuren et al., 1999) with predictive mean matching (Little, 1988; White et al., 2011). We first performed the multiple imputation by specifying the conditional model for each incomplete variable given the outcome and all other variables. We then performed a second multiple imputation procedure by specifying the conditional model for each incomplete variable given all other covariates, but excluding the outcome. For each procedure, *M* = *M*_2_ = 5 imputed datasets were created.

### 3.7. Deriving the prediction model

The multiply imputed data from the first MI procedure were used to derive the full prediction model, which included the seven variables log(cprs0+1), log(onset), log(distot), age, sex, status and centre, and up to seven partial prediction models, which included the following sets of covariates (corresponding to seven possible observed covariate patterns):

1. {log(onset), log(distot), age sex, status, centre};
2. {log(cprs0+1), log(distot), age sex, status, centre};
3. {log(cprs0+1), log(onset), age sex, status, centre};
4. {log(distot), age sex, status, centre};
5. {log(onset), age sex, status, centre};
6. {log(cprs0+1), age sex, status, centre};
7. {age, sex, status, centre}.

### 3.8. Extracting the predictions and estimating the model performance measures

Using the imputation-specific and pooled regression coefficients from the full prediction model, predictions P1–P9 were extracted. Using the imputation-specific and combined regression coefficients from the partial prediction models, predictions P1–P3 were extracted.

The predictions P1–P9 were used with the simulated outcomes to calculate imputation-specific and overall performance measures, which included the MSPE and the AUROC. Rubin's rules were applied to the imputation-specific performance measures in our “pooled performance” approach. The pooled predictions were used directly to calculate the overall performance measure in our “pooled prediction” approach.

## 4. Simulation results

### 4.1. Summary of the simulation studies

The results from the different simulation scenarios are summarized below for the linear and logistic prediction models. For comparison, we also evaluated the performance of models estimated using the simulated full-data and complete-cases analysis. Note that different prediction models are fitted and evaluated for the following approaches: (i) full-data, (ii) complete-cases, (iii) multiple imputation, and (iv) partial prediction models. We consider the evaluation on the full data to be the gold standard approach for ideal model performance and methods based on predictions P1–P4 all attempt to estimate this. The impact of optimism is minor and is addressed in the discussion. We provide results for methods based on predictions P5–P6 as a comparison. We do not advocate a gold standard approach to estimate pragmatic model performance. We report the mean and Monte Carlo errors of the performance measures over the 1000 simulated datasets.

### 4.2. Linear model

Simulation results for the estimated MSPEs are shown in Table 3. Across all approaches, there were no or minor differences in the estimated MSPEs between use of 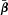 versus 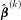 and only the results for the latter are reported. We first note that under MCAR the complete-case analyses produced MSPEs that were similar to the full-data analyses, whereas in the MAR scenarios, the MSPEs were smaller, especially for the scenario with 60% missing cprs0.

**Table 3:** Mean squared prediction errors (x100) (Monte Carlo errors) from simulated linear model. Predictions from multiply imputed data are evaluated using imputation-specific regression coefficients 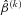 (*M* = *M*_2_ = 5)

Missing data pattern		Mean squared prediction errors (×100) (Monte Carlo errors)					
		Monotone MAR				Monotone MCAR	Independent MCAR
% missing cprs0		30%		60%		30%	30%
Coefficient for log(cprs0+1)		0.34	0.68	0.34	0.68	0.68	0.68
Model evaluated and method of evaluation							
*Model fitted and evaluated on simulated full data*		59.8 (0.1)	59.8 (0.1)	59.9 (0.1)	59.8 (0.1)	59.7 (0.1)	59.8 (0.1)
*Model fitted and evaluated on complete-cases*		57.3 (0.1)	57.8 (0.1)	55.3 (0.2)	55.4 (0.2)	59.4 (0.1)	59.4 (0.1)
Model fitted to MI dataa): Estimators of ideal model performance							
*Model evaluated on complete-cases*	Pooled performance P1	58.8 (0.1)	58.9 (0.1)	59.4 (0.2)	59.6 (0.2)	59.7 (0.1)	60.0 (0.1)
	Pooled prediction P2 = P3	58.7 (0.1)	58.8 (0.1)	59.1 (0.2)	59.2 (0.2)	59.6 (0.1)	59.9 (0.1)
*Model evaluated on MI data*a)	Pooled performance P4	59.6 (0.1)	59.5 (0.1)	60.0 (0.1)	60.7 (0.1)	59.7 (0.1)	60.3 (0.1)
	Pooled prediction P5 = P6	58.0 (0.1)	55.7 (0.1)	56.5 (0.1)	52.1 (0.1)	55.0 (0.1)	56.2 (0.1)
Model fitted to MI dataa): Estimators of pragmatic model performance							
*Model evaluated on second MI data*b)	Pooled performance P7	65.9 (0.1)	78.3 (0.1)	72.5 (0.1)	99.9 (0.3)	77.6 (0.1)	75.3 (0.1)
	Pooled prediction P8 = P9	64.0 (0.1)	72.2 (0.1)	68.0 (0.1)	85.5 (0.2)	70.3 (0.1)	69.4 (0.1)
Partial prediction models fitted to MI dataa): Estimators of pragmatic model performance							
*Models evaluated on observed covariates*	Pooled performance P1	62.4 (0.1)	67.8 (0.1)	64.9 (0.1)	75.5 (0.1)	68.4 (0.1)	67.5 (0.1)
	Pooled prediction P2 = P3	62.4 (0.1)	67.8 (0.1)	64.7 (0.1)	75.3 (0.1)	68.3 (0.1)	67.4 (0.1)

a) Imputed covariates ***x***^(*k*)^ are imputed from the set of imputation models used in deriving the prediction model.

b) Imputed covariates ***x***^(*)*)^ are imputed from a second set of imputation models which exclude the outcome variable.

Under all MAR scenarios, MSPEs using predictions P1–P3 were significantly lower (*p* < 0.001 using paired *t*-test) than those from the full-data analysis but to a lesser extent than the complete-case analysis. In contrast, under the MCAR scenarios MSPEs based on predictions P1–P3 were close to those from the full-data analysis. We note that MSPEs using P1, which incorporates between-imputation variability from 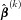, are always higher than MSPEs using P2 = P3, which in the linear model equate to using 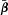.

Across all scenarios, approach P4 generally gave the closest approximation to the full data result. However, under the 60% missing cprs0 and a stronger cprs0 predictor MAR scenario, the MSPE using predictions P4 was significantly greater (*p* < 0.001 using paired *t*-test) than those from the full-data analysis. MSPEs on pooled predictions P5 = P6 were substantially smaller, wrongly suggesting better model performance, especially for the scenario with 60% missing cprs0 and a stronger cprs0 predictor. Results for P4–P6 were similar between the MCAR and MAR scenarios.

Pragmatic MSPEs were substantially larger than ideal MSPEs and also varied between our two proposed approaches. Predictions P7–P9 gave larger estimated MSPEs than those from the partial prediction models. To investigate whether these differences were due to the small number of *M* and *M_2_*, we repeated the simulation using *M* = *M_2_* = 50 and using a single second deterministic imputation for the scenario with 60% missing cprs0 and a stronger cprs0 predictor. The disparity remained, although to a lesser extent (Supporting Information Table 1). Predictions P7 gave the largest MSPEs across all scenarios, especially for the scenario with 60% missing cprs0 and a stronger cprs0 predictor. We expect this is because of the incorporated between-imputation variability from the second MI approach in P7, which was averaged out in the pooled prediction approach using P8 and P9.

### 4.3. Logistic model

The simulation results for the estimated MSPEs and AUROC for the logistic model are shown in Tables 4 and 5, respectively. Observations are similar to those from the linear model with the following exceptions. The use of 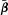 generally gave slightly lower (better) MSPE estimates and higher (better) AUROC estimates than 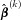 (Supporting Information Tables 2 and 3). Small differences were observed between measures based on pooled linear predictors versus pooled probabilities; we expected this finding for the AUROC measures as the estimates are based on ranks of the predictions and not their absolute values.

**Table 4:** Mean squared prediction errors (x100) (Monte Carlo errors) from simulated logistic model. Predictions from multiply imputed data are evaluated using imputation-specific regression coefficients 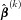 (*M* = *M*_2_ = 5)

Missing data pattern		Mean squared prediction errors (×100) (Monte Carlo errors)					
		Monotone MAR				Monotone MCAR	Independent MCAR
% missing cprs0		30%		60%		30%	30%
Prevalence of outcome		25%	8%	25%	8%	8%	8%
Model evaluated and method of evaluation							
*Model fitted and evaluated on simulated full data*		16.6 (0.02)	6.82 (0.02)	16.6 (0.02)	6.80 (0.02)	6.82 (0.02)	6.79 (0.02)
*Model fitted and evaluated on complete-cases*		14.7 (0.06)	6.46 (0.05)	13.5 (0.04)	5.28 (0.04)	6.86 (0.03)	6.90 (0.03)
Model fitted to MI dataa): Estimators of ideal model performance							
*Model evaluated on complete-cases*	Pooled performance P1	16.2 (0.07)	6.48 (0.06)	14.0 (0.04)	4.59 (0.03)	6.82 (0.03)	6.82 (0.03)
	Pooled probability P2	16.2 (0.07)	6.47 (0.06)	13.9 (0.04)	4.54 (0.03)	6.80 (0.03)	6.81 (0.03)
	Pooled linear predictor P3	16.2 (0.07)	6.46 (0.06)	13.9 (0.04)	4.54 (0.03)	6.80 (0.03)	6.81 (0.03)
*Model evaluated on MI data*a)	Pooled performance P4	16.6 (0. 02)	6.83 (0.02)	16.7 (0.02)	6.78 (0.02)	6.83 (0.02)	6.83 (0.02)
	Pooled probability P5	16.4 (0. 03)	6.69 (0.02)	16.2 (0.03)	6.55 (0.03)	6.68 (0.02)	6.74 (0.02)
	Pooled linear predictor P6	16.3 (0. 03)	6.65 (0.02)	16.2 (0.03)	6.53 (0.03)	6.68 (0.02)	6.73 (0.02)
Model fitted to MI dataa): Estimators of pragmatic model performance							
*Model evaluated on second MI data*b)	Pooled performance P7	17.6 (0. 02)	7.19 (0.02)	18.1 (0.02)	7.54 (0.03)	7.19 (0.02)	6.94 (0.02)
	Pooled probability P8	17.3 (0. 02)	7.04 (0.03)	17.5 (0.02)	7.25 (0.02)	7.01 (0.02)	6.85 (0.02)
	Pooled linear predictor P9	17.3 (0. 03)	6.99 (0.03)	17.6 (0.02)	7.30 (0.02)	7.01 (0.02)	6.94 (0.02)
Partial prediction models fitted to MI dataa): Estimators of pragmatic model performance							
*Models evaluated on observed covariates*	Pooled performance P1	17.0 (0. 02)	6.96 (0.03)	17.4 (0.02)	7.21 (0.02)	6.97 (0.02)	6.90 (0.02)
	Pooled probability P2	17.0 (0. 02)	6.94 (0.03)	17.4 (0.02)	7.19 (0.02)	6.97 (0.02)	6.98 (0.02)
	Pooled linear predictor P3	17.0 (0. 02)	6.94 (0.03)	17.4 (0.02)	7.19 (0.02)	6.97 (0.02)	6.97 (0.02)

a) Missing covariates ***x***^(*k*)^ are imputed from the set of imputation models used in deriving the prediction model.

b) Missing covariates ***x***^(*)*)^ are imputed from a second set of imputation models which exclude the outcome variable.

**Table 5:** AUROC (Monte Carlo errors) from simulated logistic model. Predictions from multiply imputed data are evaluated using imputation-specific regression coefficients 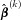 (*M* = *M*_2_ = 5)

Missing data pattern		AUROC (Monte Carlo errors)					
		Monotone MAR				Monotone MCAR	Independent MCAR
% missing cprs0		30%		60%		30%	30%
Prevalence of outcome		25%	8%	25%	8%	8%	8%
Model evaluated and method of evaluation							
*Model fitted and evaluated on simulated full data*		0.744 (0.001)	0.825 (0.001)	0.744 (0.001)	0.826 (0.001)	0.825 (0.001)	0.825 (0.001)
*Model fitted and evaluated on complete-cases*		0.789 (0.001)	0.869 (0.001)	0.773 (0.001)	0.842 (0.001)	0.827 (0.001)	0.830 (0.001)
Model fitted to MI dataa): Estimators of ideal model performance							
*Model evaluated on complete-cases*	Pooled performance P1	0.760 (0.001)	0.842 (0.001)	0.753 (0.001)	0.827 (0.001)	0.824 (0.001)	0.825 (0.001)
	Pooled probability P2	0.761 (0.001)	0.844 (0.001)	0.756 (0.001)	0.834 (0.001)	0.825 (0.001)	0.826 (0.001)
	Pooled linear predictor P3	0.761 (0.001)	0.845 (0.001)	0.757 (0.001)	0.835 (0.001)	0.825 (0.001)	0.826 (0.001)
*Model evaluated on MI data*a)	Pooled performance P4	0.742 (0.001)	0.822 (0.001)	0.739 (0.001)	0.818 (0.001)	0.821 (0.001)	0.820 (0.001)
	Pooled probability P5	0.752 (0.001)	0.829 (0.001)	0.762 (0.001)	0.843 (0.001)	0.835 (0.001)	0.828 (0.001)
	Pooled linear predictor P6	0.754 (0.001)	0.835 (0.001)	0.764 (0.001)	0.848 (0.001)	0.837 (0.001)	0.830 (0.001)
Model fitted to MI dataa): Estimators of pragmatic model performance							
*Model evaluated on second MI data*b)	Pooled performance P7	0.706 (0.001)	0.794 (0.001)	0.686 (0.001)	0.752 (0.001)	0.791 (0.001)	0.800 (0.001)
	Pooled probability P8	0.714 (0.001)	0.800 (0.001)	0.705 (0.001)	0.781 (0.001)	0.805 (0.001)	0.810 (0.001)
	Pooled linear predictor P9	0.714 (0.001)	0.804 (0.001)	0.704 (0.001)	0.776 (0.001)	0.804 (0.001)	0.809 (0.001)
Partial prediction models fitted to MI dataa): Estimators of pragmatic model performance							
*Models evaluated on observed covariates*	Pooled performance P1	0.727 (0.001)	0.808 (0.001)	0.709 (0.001)	0.785 (0.001)	0.809 (0.001)	0.805 (0.001)
	Pooled probability P2	0.728 (0.001)	0.809 (0.001)	0.711 (0.001)	0.788 (0.001)	0.810 (0.001)	0.806 (0.001)
	Pooled linear predictor P3	0.728 (0.001)	0.810 (0.001)	0.711 (0.001)	0.788 (0.001)	0.810 (0.001)	0.806 (0.001)

a) Missing covariates ***x***^(*k*)^ are imputed from the set of imputation models used in deriving the prediction model.

b) Missing covariates ***x***^(*)*)^ are imputed from a second set of imputation models which exclude the outcome variable.

## 5. Calibration slopes

Calibration is another aspect of model performance which could be assessed using multiply imputed data. One approach to calibration regresses observed outcomes on predicted outcomes (Cox, 1958). A correct set of predictions, or predictions evaluated on the same data used to estimate them, gives a calibration slope of 1.

We explore calibration with data from the UK700 trial for the prediction of satisfaction with case management at 2 years using satisfaction with case management at baseline and age as predictors (see Table 2). We restrict attention to 490 individuals with observed data on satisfaction at 2 years and baseline age, of whom 80 individuals had missing values for baseline satisfaction. We first multiply imputed (*M* = 5) the missing values using a linear imputation model with covariates of age and satisfaction at 2 years. Using all 490 individuals, we then fitted the linear prediction model for satisfaction at 2 years with covariates of age and baseline satisfaction. We extracted imputation-specific and pooled predictions using both 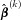 and 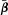 to produce P4 and P5 (as defined in Table 1), respectively. We then estimated the calibration slope on individuals with fully observed data, individuals with imputed values and all individuals. For comparison we also performed a complete-cases analysis.

Results are given in Table 6. The complete-cases analysis naturally gave the correct calibration slope. The imputation-specific calibration slopes, and consequently the pooled calibration slopes, were also naturally correct when estimated using all individuals and using 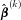. However, the pooled prediction approach overestimated the calibration slope amongst all individuals, and it is evident that this bias is due to individuals with imputed covariates as discussed in Section 70. Using predictions estimated from the pooled regression coefficients generated nonoptimal calibration slopes. Thus calibration should be assessed using 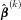 within imputed data sets.

**Table 6:** Calibration slopes estimated from complete-case analysis and a multiple imputation approach

Analysis approach	Individuals with complete data	Individuals with imputed covariates	All individuals *N* = 90
	*N* = 410	*N* = 80	
Complete-cases analysis	1.00 (0.15)	NA	NA
Multiple imputation analysis using 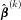 for prediction			
Imputation-specific calibration slopes			
Imputation 1	1.03 (0.17)	0.68 (0.37)	1.00 (0.15)
Imputation 2	1.03 (0.17)	0.86 (0.35)	1.00 (0.15)
Imputation 3	1.05 (0.17)	0.80 (0.35)	1.00 (0.15)
Imputation 4	1.14 (0.18)	0.50 (0.34)	1.00 (0.16)
Imputation 5	1.07 (0.16)	0.52 (0.37)	1.00 (0.16)
Pooled calibration slope	1.07 (0.18)	0.67 (0.40)	1.00 (0.15)
Calibration slope using pooled predictions	1.07 (0.17)	2.73 (0.67)	1.16 (0.16)
Multiple imputation analysis using 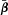 for prediction			
Imputation-specific calibration slopes			
Imputation 1	1.06 (0.17)	0.70 (0.38)	1.03 (0.15)
Imputation 2	1.06 (0.17)	0.88 (0.36)	1.03 (0.16)
Imputation 3	1.06 (0.17)	0.82 (0.36)	1.01 (0.15)
Imputation 4	1.06 (0.17)	0.47 (0.32)	0.94 (0.15)
Imputation 5	1.06 (0.17)	0.52 (0.36)	0.99 (0.15)
Pooled calibration slope	1.02 (0.18)	0.68 (0.41)	1.03 (0.15)
Calibration slope using pooled predictions	1.06 (0.17)	2.66 (0.66)	1.15 (0.16)

## 6. Discussion

In this paper we have considered the estimation and use of predictions in multiply imputed data using simulated and real data. Three main issues have been highlighted: (1) use of imputation-specific or pooled regression coefficients for estimating predictions; (2) the application of Rubin's rules to imputation-specific predictions or to model performance measures; and (3) the evaluation of the model performance in the context of future clinical settings where covariate information may be complete or partially missing.

### 6.1. Recommendations

In our simulation study and real data illustration, we observed minor differences between the use of the imputation-specific regression coefficients 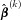 versus the pooled regression coefficients 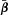. We expect important differences may occur in circumstances when 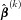 are very different for different *k*, in which case predictions based on 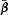 may give imprecise estimates of model performance. Importantly, predictions based on 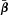 can be used to evaluate model performance for future clinical use, since it is unlikely 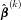 would be used in practice. Thus the choice between using 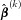 or 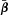 to estimate and use predictions should be justified within the context of the prediction model and the model performance evaluation.

We recommend against the use of pooled predictions calculated from the multiply imputed covariates used in model derivation. The use of such pooled predictions generally overestimates model performance. Instead, to evaluate ideal model performance for a future clinical setting where the covariates are expected to be fully observed we recommend the use of imputation-specific predictions to calculate imputation-specific measures of model performance, which may be subsequently pooled across imputations using Rubin's rules. We call this a “pool last” approach, as Rubin's rules are applied to the final estimate of interest. Guidelines for combining different performance measures, including those requiring transformation, are outlined by Marshall et al. (2009). In circumstances when the measures cannot be pooled (e.g., a calibration plot of observed against predicted outcome), we recommend reporting imputation-specific measures (e.g., a calibration plot for each imputation, or overlaying the imputation-specific calibration plots).

For future clinical settings where the patterns of missing covariates will be similar to the derivation dataset, we recommend evaluating pragmatic model performance. In general pragmatic model performance will be lower than ideal model performance. We compared two generic strategies to evaluate pragmatic model performance: (1) a multiple imputation procedure which excludes the outcome from the imputation models and (2) using partial prediction models. These two approaches evaluate different prediction models and approach (1) is sensitive to the appropriateness of the second imputation procedure while approach (2) is sensitive to the correct specification of the partial prediction models. Specifically, if missingness in the predictors is dependent on the outcome, then approach (1) will lead to poor imputations. Indeed, we additionally performed similar simulation studies to those shown for the UK700 data but under a simulated multivariate Normal scenario and observed perfect agreement between approaches (1) and (2) except when we allowed the missingness in the predictor to depend on the outcome and when we violated the multivariate normal assumption by log-transforming one of the predictors. In our real data simulations, the partial prediction models gave slightly better model performance measures than the multiple imputation methods. Other strategies exist, such as a single or multiple imputation approach which uses other easy-to-measure auxiliary variables, although we note the lack of generalizability of any single deterministic imputation for handling missing binary or categorical variables. Another less intuitive approach is to treat the outcome as a missing variable along with the missing covariates and simultaneously impute. The predicted outcome can be estimated as the average of the imputed outcomes. This method needs further investigation, but we expect it would give similar results to the partial predictions approach.

### 6.2. Further issues: Optimism correction and validation

First, we have not addressed issues of optimism correction for small samples, and this remains a topic for future research. Small amounts of optimism were evident in the simulation study: for example, the slightly smaller MSPE of the complete cases analysis than the full-data analysis in Table 3 must be due to its smaller sample size and hence greater degree of optimism. However, simulated sample sizes were large enough to make this a minor issue. In smaller samples, it would be important to allow for optimism, for example, by crossvalidation or bootstrap methods (Smith et al., 2014).

Second, we have focused on estimating and using predictions within the derivation dataset, but our approaches can also be extended to estimating and using predictions in an internal validation dataset (a different sample from the derivation dataset but within the same study population) or an external validation dataset (a sample independent of the derivation dataset (Moons et al., 2012a)). If the validation dataset has complete data then predictions P1 would be most appropriate. Often, the validation dataset will also have missing covariates, which may be handled using multiple imputation. We summarize various strategies for performing multiple imputation in derivation and validation datasets in Table 7. The choice depends largely on the validation approach. For internal validation, such as crossvalidation or split-sample validation, it may be most appropriate to draw multiple imputations in the whole dataset before separating the derivation and validation samples (approach V1 in Table 7) (O'Mahony et al., 2014). Alternatively, one could draw multiple imputations based on observed distributions of the covariates and outcomes in the derivation dataset, and apply the same set of imputation models to the validation dataset (approach V2 in Table 7). Since the imputation index *j* holds across the derivation and validation datasets, predictions P4 as defined in Table 1 can be calculated and used to evaluate *ideal* model performance. We do not recommend approaches V1 and V2 for external validation.

**Table 7:** A summary of multiple imputation approaches for derivation and validation datasets

Multiple imputation approach	Detail of multiple imputation approach	
	Derivation dataset	Validation dataset
V1. Impute simultaneously in combined dataset	Draw multiple imputations from observed distributions of covariates and outcomes in the combined derivation and validation datasets	
V2. Impute based on derivation dataset	Draw multiple imputations from observed distributions of covariates and outcomes in the derivation dataset	Draw multiple imputations from observed distributions of covariates and outcomes in the derivation dataset
V3. Impute separately	Draw multiple imputations from observed distributions of covariates and outcomes in the derivation dataset	Draw multiple imputations from observed distributions of covariates and outcomes in the validation dataset
V4. Impute separately and exclude outcome from imputation models for validation dataset	Draw multiple imputations from observed distributions of covariates and outcomes in the derivation dataset	Draw multiple imputations from observed distributions of covariates in the validation dataset

For internal or external validation, one could apply multiple imputation to the derivation and validation datasets independently (approach V3 in Table 7). Since the imputation indexes are now independent, predictions P7 are most likely to be useful. To evaluate *pragmatic* model performance, a multiple imputation approach which excludes the observed outcome could be applied to the validation dataset (approach V4 in Table 7). Partial prediction models could also be used.

### 6.3. Conclusions

In conclusion, several considerations arise when estimating and using predictions in a multiply imputed dataset. To get an overall measure of model performance for future clinical settings where individuals will have complete covariates, measures of ideal model performance should be estimated within imputed datasets, and subsequently pooled last. For a future clinical setting where individuals will have partly missing covariates, pooled predictions could be constructed using a second set of covariate imputations which make no use of observed outcomes, or partial prediction models could be assessed. The scope of these conclusions is limited by the simulation nature of the analysis and the limitation to considering linear and logistic models, but we have no reason to believe that the findings would not also apply more generally. Further work is needed to address the issues of optimism correction and how multiple imputation should be applied for internal and external validation.

## Conflict of interest

*The author has declared no conflict of interest*.
