# Supplementary material for: The estimation and use of predictions for the assessment of model performance using large samples with multiply imputed data
Source: Biom J. 2015 Jan 29;57(4):614–32. doi: 10.1002/bimj.201400004 (PMC4515100; doi:10.1002/bimj.201400004)
Supplement: Supplementary file 1 [file bimj0057-0614-sd1.zip › README file.docx]

**The estimation and use of predictions for the assessment of model performance using large samples with multiply imputed data**

Authors: Angela M Wood, Patrick Royston, Ian R White

Producer of this document and Stata Code: Angela M Wood ([amw79@medschl.cam.ac.uk](mailto:amw79@medschl.cam.ac.uk))

Software version: Stata 13.0

README file

Files:

uk500.dta data file

Linear simulation.do Stata do file to produce simulation results for the linear model, as shown in Table 3.

Logistic simulation.do Stata do file to produce simulation results for the logistic model, as shown in Tables 4 & 5.

Calibration slopes.do Stata do file to produce applied results as shown in Table 6.

Information about each available file:

1. uk500.dta

An example Stata dataset. This is a sample of 500 individuals from the original sample of 708 individuals as used in the published manuscript. A description of the data variables used in the manuscript can be found in Table 2.

The dataset should be saved into a readable folder. For the purpose of the code in the available Stata.do files, we will assume the dataset has been saved into the folder named C:.

1. Linear simulation.do

This Stata.do file can be directly executed, provided that the absolute paths for the downloaded data are correct (e.g., C:/uk500.dta).

The file creates 2 new datasets:

uk500use.dta: a complete dataset for which missing values in variables of interest are singly imputed. The data variables are the same as those in uk500.dta.

Resultslinear.dta: a postfile dataset with all the individual simulation results in. The data variables correspond to the prediction methods P1-P8 proposed in Table 1 and are explained below:

sim Simulation number

msetrue Mean Squared Errors for the model fitted and evaluated on simulated full data

msetrue Mean Squared Errors for the model fitted and evaluated on complete cases

MSE1k-MSE8k Mean Squared Errors, using predictions P1-P8 with imputation-specific regression coefficients.

MSE1bar-MSE8bar Mean Squared Errors, using predictions P1-P8 with pooled regression coefficients.

MSEPP1k-MSEPP3k Mean Squared Errors, using partial predictions P1-P3 with imputation-specific regression coefficients

MSEPP1bar-MSEPP3bar Mean Squared Errors, using partial predictions P1-P3 with pooled regression coefficients.

The provided file produces the results for the following simulation scenario: Linear model, 60% monotone MAR, stronger coefficient for log(crps0+1). The results are appended into the bottom of Linear simulation.do and are similar (but not identical due to reduced sample) to the 4^th^ column of results in Table 3. Results for the other columns in Table 3 are obtainable by making minor edits as indicated in the Linear simulation.do file.

1. Logistic simulation.do

This Stata.do file can be directly executed, provided that the absolute paths for the downloaded data are correct (e.g., C:/uk500.dta).

The file creates 2 new datasets:

uk500use.dta: a complete dataset for which missing values in variables of interest are singly imputed. The data variables are the same as those in uk500.dta.

Resultslogistic.dta: a postfile dataset with all the individual simulation results in. The data variables correspond to the prediction methods P1-P9 proposed in Table 1 and are explained below:

sim Simulation number

msetrue Mean Squared Errors for the model fitted and evaluated on simulated full data

msecc Mean Squared Errors for the model fitted and evaluated on complete cases

MSE1k-MSE8k Mean Squared Errors, using predictions P1-P8 with imputation-specific regression coefficients.

MSE1bar-MSE8bar Mean Squared Errors, using predictions P1-P8 with pooled regression coefficients.

MSEPP1k-MSEPP3k Mean Squared Errors, using partial predictions P1-P3 with imputation-specific regression coefficients

MSEPP1bar-MSEPP3bar Mean Squared Errors, using partial predictions P1-P3 with pooled regression coefficients.

roctrue Area under the ROC curve for the model fitted and evaluated on simulated full data

roccc Area under the ROC curve for the model fitted and evaluated on complete cases

R1k-R9k Area under the ROC curve, using predictions P1-P8 with imputation-specific regression coefficients.

R1bar-R9bar Area under the ROC curve, using predictions P1-P8 with pooled regression coefficients.

RPP1k- Area under the ROC curve, using partial predictions P1-P3 with imputation-specific regression coefficients

RPP1bar-RPP3bar Area under the ROC curve, using partial predictions P1-P3 with pooled regression coefficients.

The provided file produces the results for the following simulation scenario: Logistic model, 60% monotone MAR, 25% prevalence of outcome. The results are appended into the bottom of Logistic simulation.do and are similar (but not identical due to reduced sample) to the 3rd column of results in Tables 4 & 5. Results for the other columns in Tables 5 & 6 are obtainable by making minor edits as indicated in the Logistic simulation.do file.

1. Calibration slopes.do

This file reads in the uk500.dta dataset and outputs the calibration slopes as provided in Table 6. The results are not identical to those published due to reduced sample of uk500.dta.
