# Supplementary material for: The estimation and use of predictions for the assessment of model performance using large samples with multiply imputed data
Source: Biom J. 2015 Jan 29;57(4):614–32. doi: 10.1002/bimj.201400004 (PMC4515100; doi:10.1002/bimj.201400004)
Supplement: Supplementary file 2 [file bimj0057-0614-sd2.docx]

**Supplementary material**

|  | | **Mean squared prediction errors (x100) (Monte carlo errors)** |
| --- | --- | --- |
| **Missing data pattern** | | Monotone MAR |
| % missing cprs0 | | 30% |
| Coefficient for log(cprs0+1) | | 0.68 |
| ***Model fitted to MI data*: Estimators of pragmatic model performance*** | | |
| *Model evaluated on second MI data*** | Pooled performance P7 | 78.2 (0.1) |
|  | Pooled prediction P8=P9 | 70.7 (0.1) |
| *Model evaluated on deterministic imputation**** | Pooled performance P1 | 69.3 (0.1) |
|  | Pooled prediction P2=P3 | 69.3 (0.1) |
| ***Partial prediction models fitted to MI data*: Estimators of pragmatic model performance*** | | |
| *Models evaluated on observed covariates* | Pooled performance P1 | 67.8 (0.1) |
|  | Pooled prediction P2=P3 | 67.7 (0.1) |

**Supplementary Table 1: Mean squared prediction errors (x100) (Monte Carlo errors) from simulated linear model.** Predictions from multiply imputed data are evaluated using imputation-specific regression coefficients$\hat{\beta}^{(k)}$ (*M*=*M*_2_=50).

*Missing covariates *x*^(^*^k^*^)^ are imputed from the set of imputation models used in deriving the prediction model .

**Missing covariates *x*^(^*^j^*^)^ are imputed from a second set of imputation models which exclude the outcome variable.

***Missing covariates are deterministically singly imputed from a second set of imputation models which exclude the outcome variable.

|  | | **Mean squared prediction errors (x100) (Monte Carlo errors)** | | | | | |
| --- | --- | --- | --- | --- | --- | --- | --- |
| Missing Data pattern | | Monotone MAR | | | | Monotone MCAR | Independent MCAR |
| % missing cprs0 | | **30%** | | **60%** | | **30%** | **30%** |
| Prevalence of outcome | | **25%** | **8%** | **25%** | **8%** | **8%** | **8%** |
| **Model evaluated and method of evaluation** | | | | | | | |
| *Model fitted and evaluated on simulated full data* | | 16.6 (0.02) | 6.82 (0.02) | 16.6 (0.02) | 6.80 (0.02) | 6.82 (0.02) | 6.79 (0.02) |
| *Model fitted and evaluated on complete-cases* | | 14.7 (0.06) | 6.46 (0.05) | 13.5 (0.04) | 5.28 (0.04) | 6.86 (0.03) | 6.90 (0.05) |
| ***Model fitted to MI data*: Estimators of ideal model performance*** | | | | | | | |
| *Model evaluated on complete-cases* | Prediction  | 16.2 (0.07) | 7.40 (0.06) | 13.9 (0.04) | 4.50 (0.03) | 6.74 (0.03) | 6.73 (0.06) |
| *Model evaluated on MI data** | Pooled performance P4 | 16.6 (0.02) | 6.81 (0.02) | 16.8 (0.03) | 6.83 (0.02) | 6.80 (0.02) | 6.77 (0.02) |
|  | Pooled probability P5 | 16.4 (0. 03) | 6.68 (0.02) | 16.4 (0.03) | 6.62 (0.02) | 6.65 (0.02) | 6.69 (0.02) |
|  | Pooled linear predictor P6 | 16.4 (0. 03) | 6.64 (0.02) | 16.4 (0.03) | 6.62 (0.02) | 6.66 (0.02) | 6.69 (0.02) |
| ***Model fitted to MI data*: Estimators of pragmatic model performance*** | | | | | | | |
| *Model evaluated on second MI data*** | Pooled performance P7 | 17.5 (0. 02) | 7.11 (0.02) | 17.9 (0. 02) | 7.38 (0.02) | 7.11 (0.02) | 6.94 (0.02) |
|  | Pooled probability P8 | 17.3 (0. 02) | 6.98 (0.02) | 17.5 (0.02) | 7.18 (0.02) | 6.95 (0.02) | 6.85 (0.02) |
|  | Pooled linear predictor P9 | 17.3 (0.03) | 6.93 (0.02) | 17.6 (0.02) | 7.22 (0.02) | 6.95 (0.02) | 6.86 (0.02) |
| ***Partial prediction models fitted to MI data*: Estimators of pragmatic model performance*** | | | | | | | |
| *Models evaluated on observed covariates* | Prediction  | 17.0 (0. 02) | 6.87 (0.02) | 17.4 (0.02) | 7.11 (0.02) | 6.90 (0.02) | 6.90 (0.02) |

Supplementary Table 2: Mean squared prediction errors (x100) (Monte Carlo errors) from simulated logistic model. Predictions from multiply imputed data are evaluated using pooled regression coefficients$\bar{\beta}$ (*M*=*M*_2_=5).

*Missing covariates *x*^(^*^k^*^)^ are imputed from the set of imputation models used in deriving the prediction model .

**Missing covariates *x*^(^*^j)^* are imputed from a second set of imputation models which exclude the outcome variable.

|  | | **AUROC (Monte Carlo errors)** | | | | | | | |
| --- | --- | --- | --- | --- | --- | --- | --- | --- | --- |
| Missing data pattern | | Monotone MAR | | | | | | Monotone MCAR | Independent MCAR |
| % missing cprs0 | | **30%** | | | | **60%** | | **30%** | **30%** |
| Prevalence of outcome | | **25%** | | **8%** | | **25%** | **8%** | **8%** | **8%** |
| **Model evaluated and method of evaluation** | | | | | | | | | |
| *Model fitted and evaluated on simulated full data* | | 0.743 (0.001) | | | 0.825 (0.001) | 0.744 (0.001) | 0.826 (0.001) | 0.825 (0.001) | 0.825 (0.001) |
| *Model fitted and evaluated on complete-cases* | | 0.789 (0.001) | | | 0.869 (0.001) | 0.773 (0.001) | 0.842 (0.001) | 0.827 (0.001) | 0.830 (0.001) |
| ***Model fitted to MI data*: Estimators of ideal model performance*** | | | | | | | | | |
| *Model evaluated on complete-cases* | Prediction  | 0.761 (0.001) | | | 0.846 (0.001) | 0.757 (0.001) | 0.836 (0.001) | 0.827 (0.001) | 0.827 (0.001) |
| *Model evaluated on MI data** | Pooled performance P4 | 0.742 (0.001) | | | 0.822 (0.001) | 0.735 (0.001) | 0.813 (0.001) | 0.821 (0.001) | 0.820 (0.001) |
|  | Pooled probability P5 | 0.750 (0.001) | | | 0.829 (0.001) | 0.754 (0.001) | 0.834 (0.001) | 0.834 (0.001) | 0.828 (0.001) |
|  | Pooled linear predictor P6 | 0.752 (0.001) | | | 0.835 (0.001) | 0.756 (0.001) | 0.837 (0.001) | 0.835 (0.001) | 0.829 (0.001) |
| ***Model fitted to MI data*: Estimators of pragmatic model performance*** | | | | | | | | | |
| *Model evaluated on second MI data*** | Pooled performance P7 | | 0.707 (0.001) | | 0.797 (0.001) | 0.689 (0.001) | 0.759 (0.001) | 0.794 (0.001) | 0.802 (0.001) |
|  | Pooled probability P8 | | 0.714 (0.001) | | 0.802 (0.001) | 0.705 (0.001) | 0.783 (0.001) | 0.807 (0.001) | 0.812 (0.001) |
|  | Pooled linear predictor P9 | | 0.714 (0.001) | | 0.806 (0.001) | 0.704 (0.001) | 0.779 (0.001) | 0.806 (0.001) | 0.810 (0.001) |
| ***Partial prediction models fitted to MI data*: Estimators of pragmatic model performance*** | | | | | | | | | |
| *Models evaluated on observed covariates* | Prediction  | | 0.728 (0.001) | | 0.811 (0.001) | 0.711 (0.001) | 0.790 (0.001) | 0.812 (0.001) | 0.807 (0.001) |

Supplementary Table 3: AUROC (Monte Carlo errors) from simulated logistic model. Predictions from multiply imputed data are evaluated using pooled regression coefficients $\bar{\beta}$ (*M*=*M*_2_=5).

*Missing covariates *x*^(^*^k^*^)^ are imputed from the set of imputation models used in deriving the prediction model .

**Missing covariates *x*^(^*^)^*^)^ are imputed from a second set of imputation models which exclude the outcome variable.
